# Case Report: A usual procedure in an unusual situation: a patient with a rare Ehlers Danlos/osteogenesis imperfecta overlap undergoing aortic valve replacement

**DOI:** 10.3389/fcvm.2025.1480363

**Published:** 2025-02-24

**Authors:** Nicolas Nunez-Ordonez, Andrés F. Amado-Olivares, Andrés F. Jimenez-Ordonez, Carlos Obando, Tomas Chalela, Julián Senosiain, Nestor Sandoval, Jaime Camacho-Mackenzie, Carlos Villa-Hincapié

**Affiliations:** ^1^ Department of Cardiovascular Surgery, Fundacion Cardioinfantil-Instituto de Cardiologia, Bogotá, Colombia; ^2^ Faculty of Medicine, Universidad del Rosario, Bogotá, Colombia

**Keywords:** aortic valve replacement, Ehlers-Danlos, osteogenesis imperfecta, biological SAVR, genetic syndrome

## Abstract

Connective tissue disorders are known to cause cardiac and vascular complications. We present the case of a 37-year-old female patient with a rare Ehlers Danlos/Osteogenesis Imperfecta Overlap Syndrome, referred to cardiac surgery with aortic valve regurgitation, who underwent a successful Biological Surgical Aortic Valve Replacement (SAVR). A multidisciplinary, patient-centered, heart-team approach is essential in managing patients with rare genetic disorders to optimize postoperative outcomes. Adult cardiac surgeons must become familiar with genetic syndromes and their implications for improving perioperative outcomes.

## 1 Introduction

Ehlers-Danlos Syndrome (EDS) encompasses a group of connective tissue disorders related to mutations of genes encoding collagen I (1). Cardiac-valvular EDS is a rare variant that shares some of the most common characteristics of classic EDS (Joint hypermobility, skin hyperextensibility) that often requires heart surgery due to severe valvular compromise (2, 3).

We describe a case of an aortic valve replacement due to severe aortic regurgitation in a young patient with a recently described (1) Osteogenesis imperfecta/Ehlers Danlos overlap syndrome.

## 2 Case presentation

A 37-year-old female patient was referred for cardiac surgical consultation with an incidental finding of a moderate to severe aortic regurgitation. Upon first consultation the patient had no symptoms related to cardiac disease. The patient had a prior diagnosis of type I Osteogenesis Imperfecta (OI) at birth that had required multiple interventions due to severe Musculo-skeletal and ocular defects (elbow reconstruction, cornea transplant, spine reconstruction due to severe scoliosis and spondylolisthesis, and ankle osteosynthesis).

### 2.1 Physical examination

Physical examination revealed a patient with a high palate, an elevated nasal bridge, low-implantation ears, blue sclerae, hypermobility in the fingers and knee joints and hyper-elastic skin. Skeletal deformities consistent with the medical history were evident. Cardiovascular evaluation was positive for a grade III/VI holodiastolic murmur best heard at the right 2nd intercostal space.

### 2.2 Additional studies

**Figure 1:**
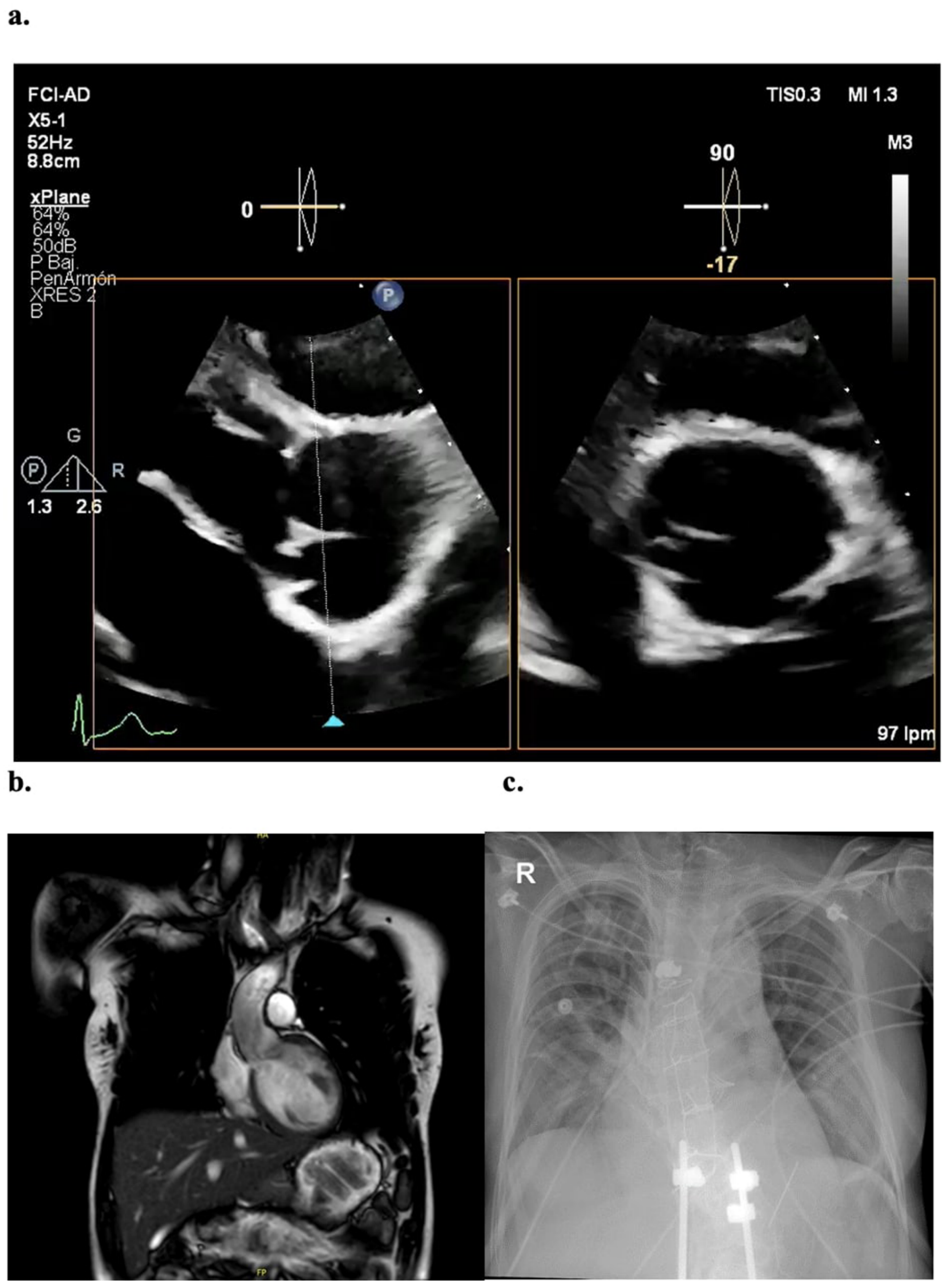
Imaging studies **(a)** preoperative transthoracic echocardiogram, with severe aortic regurgitation associated to a non-coronary cusp retraction. **(b)** Coronal view of Cardiac MRI, showing a non-dilated ascending aorta with maximum commissural diameter of 30 mm **(c)** postoperative x-ray evidencing 6 surgical steel wire sutures and sternal hemi cerclage.

Preoperative echocardiogram (Figure 1a) showed a severely dilated left ventricle with eccentric hypertrophy, LVEF 60%, and severe aortic regurgitation. The aortic root measured 36 mm. Cardiac MRI confirmed the findings with a regurgitant fraction of 59%. The ascending aorta was measured at 30 × 30 × 30 at the highest diameter (commissural level) (Figure 1b). Ergospirometry revealed no limitations in cardiovascular or pulmonary function.

### 2.3 Genetic evaluation

Given the history of OI, a genetic consultation was requested as part of the workup for Heart Team evaluation. The genetic history revealed one sister with a confirmed diagnosis of type I OI, while no other close relatives had a genetic diagnosis. A genetic sequencing was positive for a heterozygotic mutation of COL1A1 c. 572G>A (p. Gly191Asp.). The final genetic evaluation considered that the patient exhibited findings suggestive of a rare, recently described OI/EDS overlap syndrome: the genetic sequencing showed characteristic glycine residue substitutions on the type I collagen gene and some clinical features (such as blue sclerae, bone fragility or osteopenia) that were compatible with OI; on the other hand, additional findings like the cardiac-valvular compromise, soft-tissue fragility or the joint and skin hypermobility (combined with the identified mutations on the type I collagen gene) suggested the EDS spectrum.

### 2.4 Procedure

**Figure 2:**
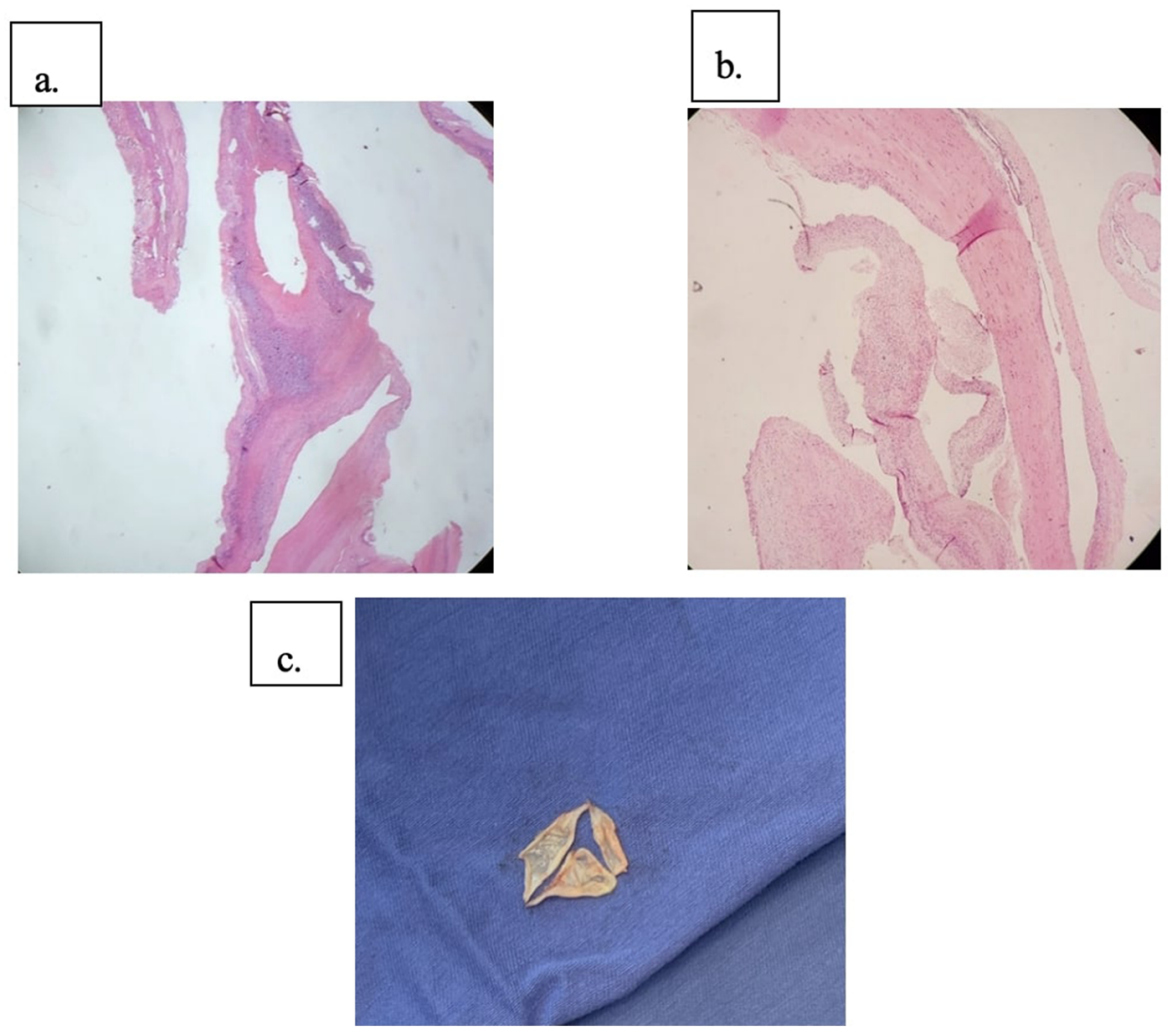
Pathologic findings **(a)** hematoxylin eosin stain showing degenerative changes in valvular stroma. **(b)** Alcian blue stain, blue-stained areas that correspond to mucin deposits **(c)** surgical specimen showing a tricuspid aortic valve with retraction of the non-coronary cusp.

After multidisciplinary discussion and agreement with the patient, an elective aortic valve replacement via median sternotomy was decided. Hyperlaxity of all soft tissues was evident and a severely fragile sternal bone with multiple previous fractures was noted. A conventional technique of arterial cannulation in the distal ascending aorta and venous cannulation through the right atrial appendage were used with a left-cavity venting through the right superior pulmonary vein. TEE was used to assess adequacy of the cannulation before entering on bypass. Notably fragile tissues were encountered on these structures and therefore minimal traction and tissue manipulation were sought. A tricuspid aortic valve was found, exhibiting a severe retraction of the non-coronary cusp (Figure 2c). The aortic root and ascending aorta were not dilated. Coronary ostia showed usual disposition. A biological *Edwards Lifesciences INSPIRIS RESILIA* 25 mm aortic valve was implanted without complications, and intraoperative TEE confirmed an adequate functioning valve with normal biventricular systolic function. Special care was taken during sternal closure due to severe frailty of the tissues. A sternal hemi-cerclage was performed, and 6 surgical steel simple sutures were placed to prevent sternal instability or dehiscence (Figure 1c). Clamp time was 72 min, and CPB time was 91 min.

Postoperative recovery was uneventful, early extubation upon arrival to the ICU was achieved. The patient was taken to the general ward after 3 days of ICU monitoring and was discharged home on POD6. Early postoperative TTE indicated a normofunctional prosthesis (peak vel. 1, 8 m/s, peak gradient 12 mmHg, mean gradient 6 mmHg).

Pathological examination of the excised tissue revealed non-specific degenerative changes in the valve stroma with mucin deposits above the valvular tissue (Figures 2a,b).

### 2.5 Follow-up

At the 12 month postoperative evaluation the patient was asymptomatic, with no signs of postoperative complications; no wound or sternal complications were noted, and a normofunctional valve with normal LV function on 3-month control TTE was confirmed.

## 3 Discussion

With recent advances in diagnostic and therapeutic techniques, patients with diverse genetic conditions have become increasingly common in adult cardiac surgery practice. It is therefore important for adult cardiac surgeons worldwide to become familiar with the challenges that each condition may impose on otherwise common surgical procedures.

We presented a case of a patient with a rare OI/EDS overlap. This is, to the author's knowledge, the first reported experience of a patient with such condition undergoing cardiac surgery. We provide an overview on the relevant characteristics of this COL-1 related disorder for the cardiac surgeon and some insights on the perioperative considerations to improve outcomes.

### 3.1 The syndrome

EDS refers broadly to a group of connective tissue disorders related mainly to mutations of the genes encoding collagen (2), manifesting with joint hypermobility, skin hyperextensibility and soft tissue and vascular fragility (1). Patients with COL1A2 mutations result in absent/reduced proα2 chains with a resultant inability to adequately produce the protein. Cardiac-valvular EDS is a rare variant of the disease (4). Patients with Cardiac-valvular EDS present with classical EDS features such as joint hypermobility, skin hyperextensibility while also developing cardiac valvular disease. These patients frequently develop mitral valve disease (usually mitral valve prolapse) but aortic valve compromise is not uncommon (2).

Osteogenesis imperfecta, on the other hand, is an autosomal dominant disease (5) that results from quantitative or qualitative defects in type I collagen. Most mutations are related to COL1A1 and COL1A2 genes. Patients frequently present with bone fragility, blue sclera, dental fragility, hearing abnormalities and hyperlaxity. Type I collagen is also a structural component in cardiac and vascular extracellular matrix making these patients prone to cardiovascular manifestations (although infrequent). Among those, aortic regurgitation due to leaflet dysfunction as in this case, has been reported (6, 7), but mitral compromise seems to be more common.

A recently described COL1-related disorder manifesting as an OI/EDS overlap (1) was diagnosed in this patient. This is an extremely rare combination reaching an incidence around 1/1,000.000 (8). Even if proper diagnostic criteria have not been established so far, the phenotype of this patient is similar to what has been reported elsewhere (8). A characteristic glycine substitution was identified in this case which could be responsible for the clinical presentation.

### 3.2 Surgical considerations

As seen in this case, patients with collagen-related disorders also require common cardiac surgical interventions, such as valvular replacement surgery or aortic root/ascending aorta surgery. However, several critical concerns are worth highlighting from this case: major issues were anticipated with handling of the weak, friable tissues; a high intra- and postoperative bleeding risk was recognized, and difficulties with sternal opening and closure were expected as well. Other potential complications may be related to a higher risk of perioperative arrhythmias due to a possible involvement of the cardiac conduction system as a part of the syndrome (6). Long-term risk such as valve dehiscence or aortic dissection should be kept into account however, a careful surgical procedure may mitigate these concerns.

Patients with collagen-related disorders have high reported mortality rates. One recent study reported a perioperative mortality exceeding 20%, mainly related to bleeding complications (6). Therefore, additional measures must be taken when caring for these types of patients.

Adequate surgical planning should include the selection of the optimal timing for the intervention, while minimizing the presence of other risk factors. In this case, the patient was being followed as an outpatient and surgery was decided in the presence of mild symptoms and guided primarily by imaging criteria given that it was considered the best scenario for a safe surgical procedure as the patient was in an overall good general health condition.

### 3.3 Intraoperative considerations

Multiple special concerns should be considered when taking care of patients with COL1-related disorders. From anesthetic care (including the risk of temporo-mandibular joint dislocation) to a higher chance of organ rupture or inducing vessel injuries with surgical handling, these patients need special attention during the intraoperative period (4, 6). Surgical planning incorporates all these aspects and raising awareness on the whole surgical team on these multiple issues is essential.

Gentle tissue handling and even including less invasive approaches (such as mini sternotomies) is recommended for these procedures (9). In this case, however, a full sternotomy was performed due to the treating surgeon's preference as it was believed that the conventional cannulation and surgical technique were safer and effective while reducing the risk of retrograde aortic dissection associated with femoral cannulation. Our approach included a preoperatively planned preventive sternal cerclage with surgical steel which proved effective in preventing sternal dehiscence at 12 months.

### 3.4 Ideal valvular prosthesis

Choosing the most appropriate valvular prosthesis is a critical decision for these patients. Current guidelines by the ACC/AHA recommend considering a mechanic aortic prosthesis in patients younger than 50 years of age (class of recommendation 2a) (10). However, in accordance with both the AHA/ACC and ESC/EACTS current guidelines (10, 11), this patient was deemed at high bleeding risk due to comorbidities; therefore, a bioprosthesis was chosen. Additionally, considering the patient's medical history, it was likely that future surgical interventions (both cardiac or non-cardiac) would be necessary, making the bioprosthesis a risk-reducing option.

A recent review by Dimitrakakis et al. on the ideal prosthesis for patients with osteogenesis imperfecta showed that bioprosthetic valves tend to yield better outcomes as compared to mechanical valves. Since these patients tend to have a high bleeding risk due to friable tissues, platelet dysfunction and capillary fragility, bioprosthetic valves seem to be a reasonable choice in terms of controlling bleeding complications. Besides, the implantation of mechanical valves may generate more mechanical trauma to the weakened, friable tissues, thus increasing the risk of aortic dissection and paravalvular leaks (5).

### 3.5 Postoperative care

In this case routine postoperative care was provided and guaranteed an uneventful recovery. Special care and awareness should be kept on the perioperative bleeding risk.

Another concern that should guide postoperative care is a delayed wound healing that is frequent in this subset of patients. From soft tissue to sternal healing, providers should be aware and anticipate the possible wound complications that could develop in the postoperative course.

### 3.6 Take-away lessons

A multidisciplinary, patient-centered, heart-team approach provides is fundamental in decision making related to perioperative care of patients with rare genetic disorders for improving outcomes.

Advances in diagnostic and therapeutic methods has led to an increase in patients with diverse genetic disorders that undergo cardiac surgery, challenging adult cardiac surgeons to become familiar with characteristics of each syndrome and its implications for improving perioperative outcomes.

## Data Availability

The original contributions presented in the study are included in the article/Supplementary Material, further inquiries can be directed to the corresponding author.

## References

[1] Morlino S Micale L Ritelli M COL1-related overlap disorder: a novel connective tissue disorder incorporating the osteogenesis imperfecta/Ehlers-Danlos syndrome overlap. Clin Genet. (2020) 97(3):396–406. 10.1111/cge.13683 31794058

[2] Brady AF Demirdas S Fournel-Gigleux S The Ehlers-Danlos syndromes, rare types. Am J Med Genet C Semin Med Genet. (2017) 175(1):70–115. 10.1002/ajmg.c.31550 28306225

[3] Guarnieri V Morlino S Di Stolfo G Mastroianno S Mazza T Castori M. Cardiac valvular Ehlers-Danlos syndrome is a well-defined condition due to recessive null variants in COL1A2. Am J Med Genet A. (2019) 179(5):846–51. 10.1002/ajmg.a.61100 30821104

[4] Azanza DXC Munín MA Sánchez GA Spernanzoni F. Prolapse and regurgitation of the four heart valves in a patient with Ehlers-Danlos syndrome: a case report. Eur Heart J Case Rep. (2019) 3(2):1–8. 10.1093/EHJCR/YTZ052 31449614 PMC6601149

[5] Dimitrakakis G Challoumas D Von Oppell UO. What type of valve is most appropriate for osteogenesis imperfecta patients? Interact Cardiovasc Thorac Surg. (2014) 19(3):499–504. 10.1093/icvts/ivu152 24876219

[6] Lamanna A Fayers T Clarke S Parsonage W. Valvular and aortic diseases in osteogenesis imperfecta. Heart Lung Circ. (2013) 22(10):801–10. 10.1016/j.hlc.2013.05.640 23791715

[7] Bonita RE Cohen IS Berko BA. Valvular heart disease in osteogenesis imperfecta: presentation of a case and review of the literature. Echocardiography. (2010) 27(1):69–73. 10.1111/j.1540-8175.2009.00973.x 19725849

[8] Morabito LA Allegri AEM Capra AP Osteogenesis Imperfecta/Ehlers-Danlos overlap syndrome and neuroblastoma-case report and review of literature. Genes (Basel). (2022) 13(4):581. 10.3390/genes13040581 35456387 PMC9024599

[9] Izzat MB Wan S Wan IYP Khaw KS Yim APC. Ministernotomy for aortic valve replacement in a patient with osteogenesis imperfecta. Ann Thorac Surg. (1999) 67(4):1171–3. 10.1016/S0003-4975(99)00126-5 10320279

[10] Otto CM Nishimura RA Bonow RO 2020 ACC/AHA guideline for the management of patients with valvular heart disease: a report of the American College of Cardiology/American Heart Association joint committee on clinical practice guidelines. Circulation. (2021) 143(5):E72–227. 10.1161/CIR.0000000000000923 33332150

[11] Vahanian A Beyersdorf F Praz F 2021 ESC/EACTS guidelines for the management of valvular heart disease. Eur Heart J. (2022) 43(7):561–632. 10.1093/eurheartj/ehab395 34453165

